# MCPIP3/Regnase-3 binds 14-3-3 proteins and contributes to the regulation of the cell cycle in human immortalized keratinocytes

**DOI:** 10.1038/s41598-025-18468-y

**Published:** 2025-09-26

**Authors:** Agata Lichawska-Cieslar, Weronika Szukala, Leopold Eckhart, Jolanta Jura

**Affiliations:** 1 Faculty of Biochemistry, Biophysics and Biotechnology, Department of General Biochemistry, Jagiellonian University, Gronostajowa 7, 30-387 Krakow, Poland; 2 Doctoral School of Exact and Natural Sciences, Jagiellonian University, Lojasiewicza 11, 30-348 Krakow, Poland; 3 Department of Dermatology, Medical University of Vienna, Währinger Gurtel 18- 20, 1090 Vienna, Austria

**Keywords:** MCPIP3, Regnase-3, Keratinocytes, Proteomics, RNA metabolism, Protein-protein interaction networks

## Abstract

Skin homeostasis is ensured by a fine-tuned balance of proliferation and differentiation of keratinocytes, disturbances in which may initiate or promote skin diseases. The monocyte chemotactic protein-induced protein 3 (MCPIP3), or Regnase-3, is a ribonuclease belonging to the MCPIP family. Keratinocyte-specific loss of MCPIP3 results in increased expression of genes related to cell division, accelerated epidermal proliferation rate, and abnormal differentiation. The aim of this study was to gain insights into the mechanisms by which MCPIP3 affects keratinocyte biology. Immunoprecipitation-proteomics was applied to identify putative interactors of MCPIP3 in HaCaT keratinocytes. It revealed that MCPIP3 forms complexes with keratin 14, 14-3-3 proteins and modulators of cell polarity. Silencing of MCPIP3 and keratin 14 led to significantly increased expression of S/G2 and G2/M phase markers, namely cyclin A2, cyclin B1 and histone H3 Ser10 phosphorylation. Simultaneous silencing of both genes had synergistic effect. Double thymidine block and release protocol was used to synchronize HaCaT cells and indicated that MCPIP3 reaches the highest level of expression in peri-mitotic cells. Altogether, our results uncover the significance of studying the post-transcriptional regulation of gene expression in the context of cell cycle related events, clarifying the mechanisms by which MCPIP3 modulates proliferation of keratinocytes.

**Supplementary Information:**

The online version contains supplementary material available at 10.1038/s41598-025-18468-y.

## Background

Keratinocytes are the major cell type building human epidermis, which undergo coordinated processes of division and differentiation. They proliferate in the basal layer, then commit to differentiate and migrate towards the surface of the epidermis^1^. On the molecular level, basal keratinocytes highly express keratin 5 (KRT5) and keratin 14 (KRT14). This pattern is then switched to keratin 1 (KRT1) and keratin 10 (KRT10) as cells commit to differentiation^2^. During terminal differentiation, filaggrin (FLG), involucrin (IVL), and transglutaminase-1 (TGM1) are highly expressed and play essential role in the formation of the protective skin barrier^3^. Mechanistically, the KRT5-KRT14 intermediate filament (IF) pair is essential for the maintenance of the structural integrity of the basal layer, whereas the KRT1-KRT10 one supports the structure of the suprabasal epidermis^4,5^. Disturbed expression of those keratin genes is a hallmark of many skin diseases, particularly those involving altered proliferation and differentiation of keratinocytes^6^.

We recently demonstrated that monocyte chemotactic protein-induced protein 3 (MCPIP3), also known as Regnase-3 modulates processes related to keratinocyte proliferation and differentiation^7^. MCPIP3 is encoded by the *ZC3H12C* gene and has RNase properties. It belongs to the MCPIP family of CCCH-type zinc finger proteins, acting as endonucleases and degrading specific mRNAs, thereby regulating gene expression post-transcriptionally^8–10^. The MCPIP family, especially MCPIP1, is central to immune regulation, inflammation resolution, antiviral defense, and tissue homeostasis^11,12^. While MCPIP1 is well-studied, the functions of MCPIP2–4 still require further investigation to fully elucidate their roles in health and disease.

MCPIP1 and MCPIP3 proteins have been identified as regulators of epidermal biology^13–16^. Loss of MCPIP1 in mouse epidermis upregulates the levels of transcripts related to inflammation and keratinocyte differentiation, leading to the development of local and systemic inflammation^13^. Our recent studies utilizing a mouse model with keratinocyte-specific deletion of MCPIP3 indicated that the activity of MCPIP3 in keratinocytes is also essential for the maintenance of proper epidermal structure and function, but the mechanism is completely different^7^. In contrast to MCPIP1-specific deletion, MCPIP3 keratinocyte-specific knockout mice did not develop skin inflammation. However, their epidermis showed elevated proliferation rate. On the molecular level, increased expression level of key factors promoting nuclear division was observed in their skin. In vitro studies on human keratinocytes showed that silencing of MCPIP3 slightly increases their viability suggesting an impact of MCPIP3 on cell cycling/proliferation^7^.

Unperturbed activity of MCPIP3 in keratinocytes is important for the proper control of their proliferation and differentiation program. When this process is unbalanced, it can lead to the development of skin diseases. The aim of the current study was to explore the mechanisms of MCPIP3-dependent effects on keratinocytes, with the focus on identification of its binding partners using immunoprecipitation-proteomics approach and investigating its role in cell cycle progression.

## Materials and methods

### Cell culture

Human immortalized HaCaT keratinocytes^17^ were cultured in high glucose DMEM supplemented with 10% fetal bovine serum (FBS, Sigma-Aldrich, St. Louis, MO, USA). The cells were grown at 37 °C and 5% CO_2_ humidified atmosphere.

### Transfection

HaCaT cells were seeded at a density of 8 × 10^5^ cells per well of a 6-well plate 24 h prior to transfection with 20 nM siGENOME Human ZC3H12C (#85463) SMARTpool, siGENOME Human KRT14 (#3861) SMARTpool or siGENOME Non-Targeting siRNA Pool #1 (Dharmacon) using JetPrime reagent (Polypus, London, UK) and collected after 72 h for RT-qPCR, western blot, flow cytometry or immunofluorescence analyses.

### Cell synchronization

HaCaT cells were seeded at 40% confluency and treated with 2 mM thymidyne (Sigma-Aldrich) for 24 h. The cells were then washed with PBS and cultured in complete DMEM for 12 h prior to addition of 2 mM thymidine for 24 h. The cells were then washed and harvested at specific intervals for flow cytometry or western blot analyses.

### Immunoprecipitation

The doxycycline-dependent TetON system was used (pLIX vectors) to obtain HaCaT cells overexpressing 3xFLAG-MCPIP3, or empty control, as described previously^18^. In parallel, HaCaT cells overexpressing 3xFLAG-MCPIP1, or empty control, were used as an experimental control (data not shown). To induce expression of exogenous protein, cells were stimulated with doxycycline (BioShop, Burlington, Canada). For immunoprecipitation, cells grown at sub-confluency were lysed in IP lysis buffer (0.5% NP-40, 150 mM NaCl, 50 mM Tris pH 7.5 with protease and phosphatase inhibitors). For mass spectrometric analysis, protein lysates were incubated with anti-FLAG M2 (Sigma-Aldrich) coated Protein G Dynabeads (Invitrogen, Darmstadt, Germany) for 2 h at 4 °C, followed by competitive elution with 1.5 mg/ml 3xFLAG peptide (Invitrogen). Eluates were snap-frozen and stored at −80 °C. For western blot, the beads were mixed with sample buffer and boiled for 5 min at 65 °C to elute proteins.

### Mass spectrometry analysis

Mass Spectrometry analysis was performed by the Core Facility for Proteomics at Medical University of Vienna. The mass spectrometry proteomics data have been deposited to the ProteomeXchange Consortium via the PRIDE^19^ partner repository with the dataset identifier PXD067727.

Immunoprecipitation eluates, prepared in triplicates, for 3xFLAG-MCPIP3 *vs*. experimental control and for 3xFLAG-MCPIP1 *vs*. experimental control (data not shown) were subjected to Mass Spectrometry analysis. The samples were reduced, alkylated and bound to the SP3 beads (GE Healthcare, Amersham, UK). Next, they were subjected to on-bead digestion with trypsin/LysC Mix (Promega, Walldorf, Germany) overnight at 37 °C in 50 mM ammonium bicarbonate, pH 8.5 (Sigma-Aldrich). After elution peptides were desalted using Pierce Peptide Desalting spin columns (Thermo Fisher Scientific, Waltham, MA, USA). The elutions were dried in a vacuum concentrator and reconstituted in 0.1% trifluoroacetic acid. LC-MS was performed on an Ultimate 3000 RSLC nano coupled directly to an Exploris 480 with FAIMSpro (all Thermo Fisher Scientific). MS scans were performed in the range from *m*/*z* 375–1650 at a resolution of 60,000 (at *m*/*z* = 200). MS/MS scans were performed choosing a resolution of 15,000; normalized collision energy of 29%; isolation width of 1.4 *m/z* and dynamic exclusion of 90s. Two different FAIMS voltages were applied (−40 V and − 60 V) with a cycle time of 1.5 s per voltage. The acquired raw MS data files were processed and analyzed using ProteomeDiscoverer (v2.4.0.305, Thermo Fisher Scientific). Precursor ion quantification was done using the Minora Feature Detector node. Only unique peptides were used for quantification, which was based on intensity. Normalization was done on total peptide amount and scaling mode on all average. Only peptides and proteins with FDR < 0.01 were reported and single peptide IDs were excluded from the dataset. Testing for differentially regulated proteins was performed using R (v4.2.0) and the R-package “limma”^20^. P-values were corrected for multiple hypothesis testing using the BH-method. Only proteins having at least 1 abundance value available per treatment-group were used for analysis. The functional annotation of genes coding differentially expressed proteins (DEPs) was performed using the R package ClusterProfiler (version 4.4)^21^. Volcano plots and dot plots were created using the ggplot2 libraries in R. The STRING database (version 12.0) was used to analyse protein-protein interaction networks and perform functional enrichment analyses^22^.

### Isolation of RNA and quantitative PCR

Total RNA was isolated using standard Fenozol (A&A Biotechnology, Gdansk, Poland) and choloroform extraction. 1 µg of RNA was reverse transcribed using oligo(dT) primer and M-MLV reverse transcriptase (Promega). Quantitative PCR was performed in duplicates on QuantStudio 3 (Thermo Fisher Scientific) using SYBR-Green based master mix (A&A Biotechnology) and gene-specific primer pairs (Sigma-Aldrich). Sequences of primers are listed in Supplemental Table S1.

## Western blot analysis

Cells were lysed in IP buffer or RIPA buffer supplemented with protease and phosphatase inhibitors. Insoluble proteins were removed by centrifugation. The BCA protein assay was used to measure protein concentration. Protein separation was carried out using 8%, 12% or 15% polyacrylamide gel electrophoresis, which was followed by transfer to nitrocellulose membrane (Merck Millipore, Billerica, MA, USA). Membranes were blocked in 5% non-fat milk and incubated with appropriate primary and secondary antibodies listed in the Supplemental Table S2, according to the manufacturer’s specifications. ChemiDoc imaging system with ImageLab software (Bio-Rad, Hercules, CA, USA) was used for protein detection and densitometric quantification.

## Immunofluorescence

For immunofluorescence staining, cells were washed with PBS and fixed with 4% paraformaldehyde/PBS (ChemCruz, Dallas, TX, USA) for 15 min at RT. Then, the cells were blocked in 5% BSA/0.3% Triton-X100/PBS for 1 h at RT and incubated with primary antibody diluted in 1% BSA/0.3% Triton-X100/PBS overnight at 4 °C. The next day, the cells were washed with PBS and incubated with secondary antibody and Hoechst 33258 (Thermo Fisher Scientific) for 1 h at RT, rinsed with dH_2_O and mounted with Prolong Glass Antifade Mountant (Invitrogen). The cells were visualized under a Leica DMC5400 fluorescence microscope (Leica Microsystems, Wetzlar, Germany). All figures were prepared and counted using ImageJ Fiji^23^.

### Flow cytometry

Ethanol-fixed cells were stained using FxCycle™ PI/RNAse Staining Solution (Invitrogen) and analysed on Attune NxT flow cytometer (Thermo Fisher Scientific).

### Statistical analysis

All the calculations and statistical analyses (except MS data) were performed using GraphPad Prism 8.0 (GraphPad, La Jolla, CA, USA). One-way or two-way ANOVA were used to analyze data with *P < 0.05, **P < 0.01, ***P < 0.001, and ****P < 0.0001. All the figures were created using CorelDraw 2024 (Corel Corporation, ON, Canada).

## Results

### MCPIP3 interacts with KRT14, 14-3-3σ and other regulators of keratinocyte proliferation and differentiation

**Fig. 1:**
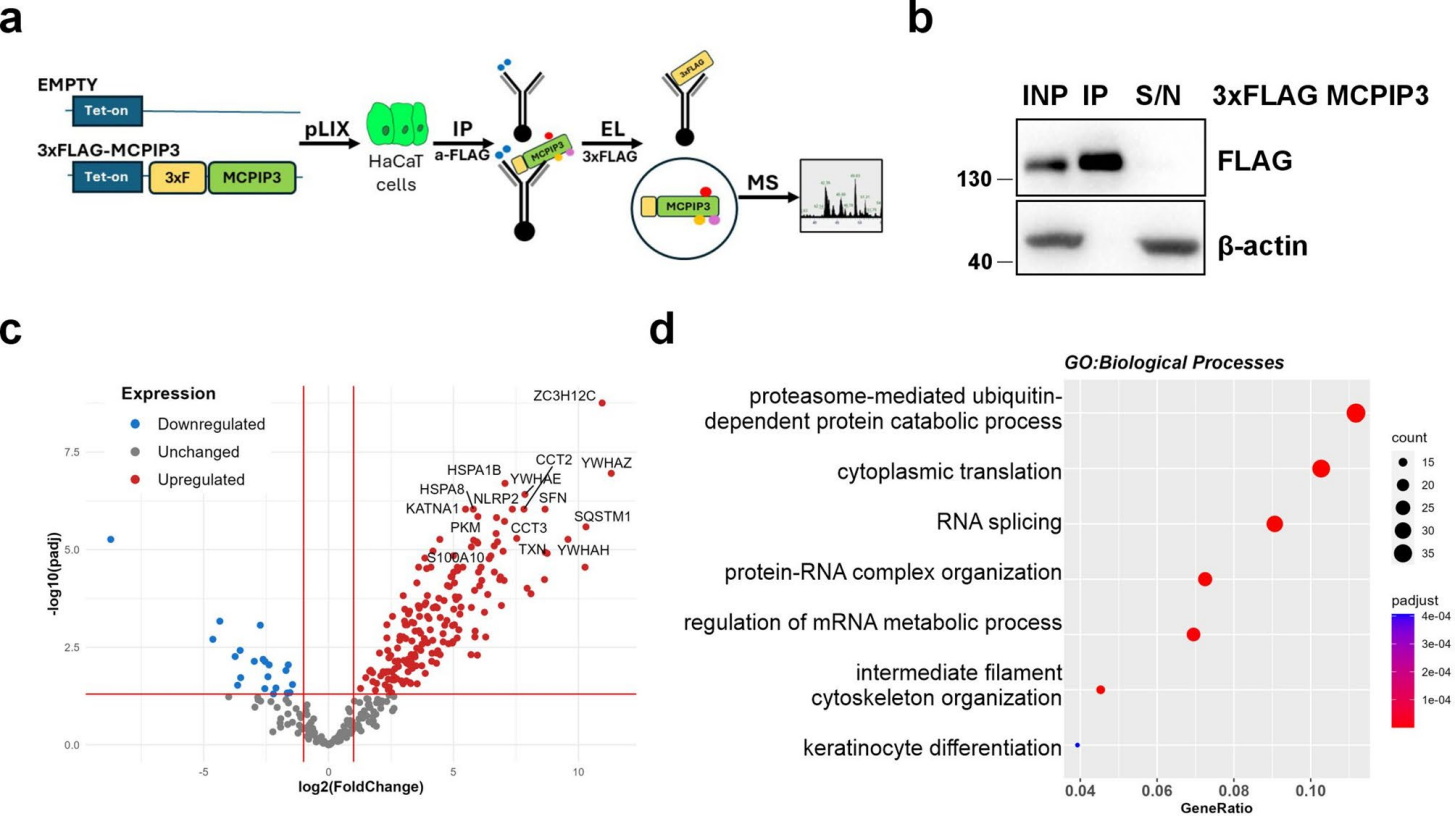
MS analysis of MCPIP3 protein complexes. **a** A scheme illustrating experimental pipeline. **b** Representative western blot analyses for FLAG and β-actin of input (INP), immunoprecipitated (IP) and unbound fraction (S/N). **c** Volcano plot representing genes coding the differentially expressed proteins (DEPs) between Empty control and 3xFLAG-MCPIP3 immunoprecipitates. Adjusted P value (padj) < 0.05, fold change (FC) > 2.0. **d** Dot plot represents selected pathways in Gene Ontology (GO) Biological Processes (BP). INP–input; IP–immunoprecipitation; EL-elution; MS–mass spectrometry S/N–supernatant.

**Fig. 2:**
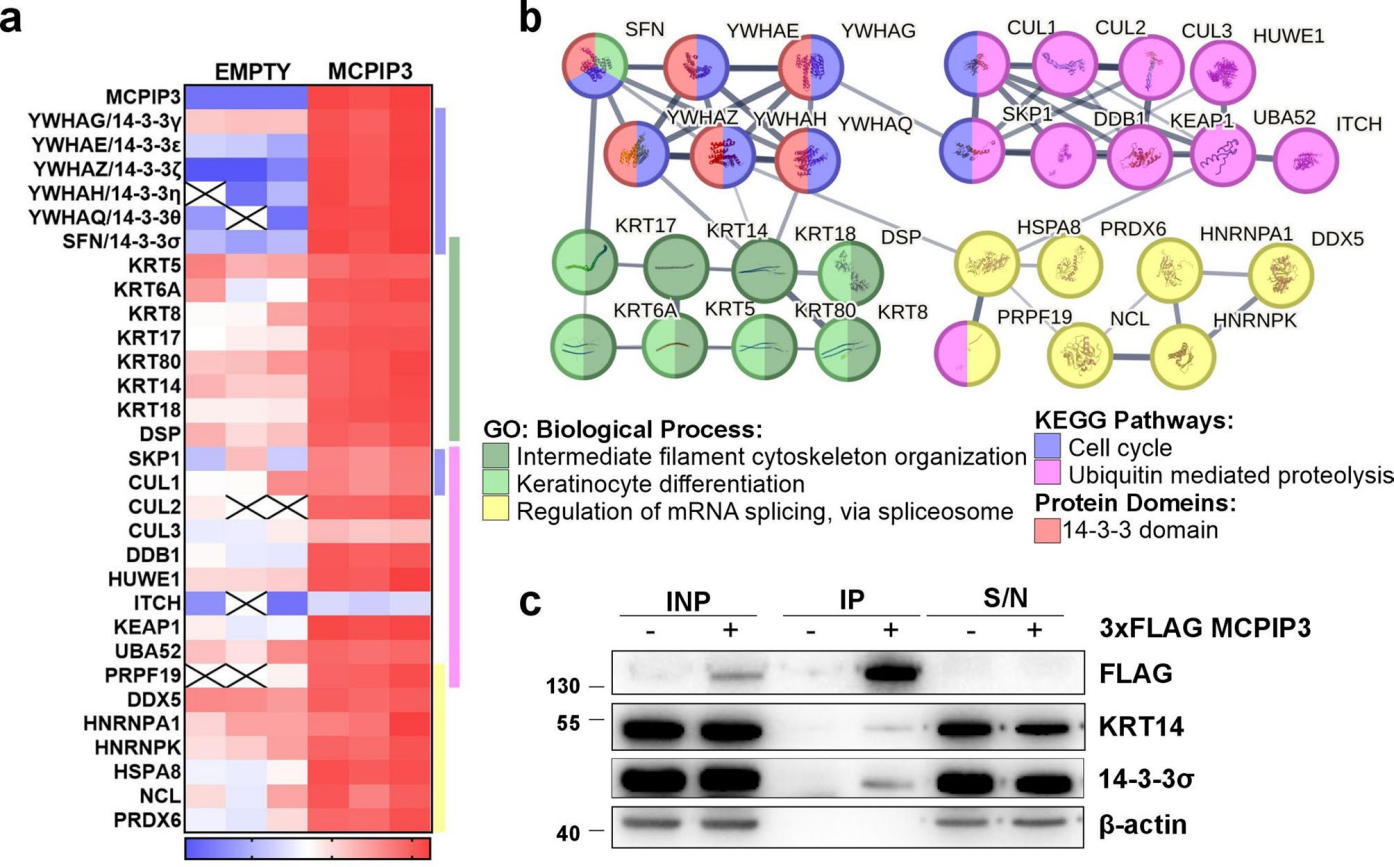
In HaCaT cells, MCPIP3 forms complexes with KRT14 and 14-3-3 proteins.** a** Heatmap representing log2 scaled abundances of selected DEPs identified in MS analysis of 3xFLAG-MCPIP3 immunoprecipitates. X – not detected. **b** Scheme represents the STRING functional analysis of protein-protein interactions. Each node represents the physical subnetwork and the thickness of line indicates the strength of data support. **c** Representative IP–western blot of analysis for FLAG, KRT14, 14-3-3σ and β-actin.

To obtain mechanistic understanding of MCPIP3 functions in keratinocytes, we performed immunoprecipitation-mass spectrometry (IP-MS) analysis using HaCaT cells with inducible expression of 3xFLAG-MCPIP3. We performed immunoprecipitation using anti-FLAG coated beads, followed by elution *via* FLAG peptide competition (Fig. 1a, b). The eluates were subjected to MS analysis, which revealed 186 proteins as significantly enriched in MCPIP3 immunoprecipitates compared to that of the control cells, with padj < 0.05 and fold change > 2.0 (Fig. 1c and Supplemental Table S3). Gene Ontology enrichment analysis revealed groups of proteins functionally related to the proteasome-mediated ubiquitin-dependent process, protein-RNA complex organization and regulation of mRNA metabolic process, which are consistent with the function of MCPIP3 as an RNase. We also identified proteins assigned to the terms of intermediate filament cytoskeleton organisation and keratinocyte differentiation (Fig. 1d), of which examples are KRT5, KRT14 and Desmoplakin/DSP (Fig. 2a, b). The list of putative interactors of MCPIP3 also included members of the 14-3-3 protein family: 14-3-3γ/YWHAG, 14-3-3ε/YWHAE, 14-3-3ζ/YWHAZ, 14-3-3η/YWHAH, 14-3-3θ/YWHAQ and 14-3-3σ/SFN, which has been assigned into KEGG category of the cell cycle (Fig. 2a, b). The STRING database indicated the interaction network between these proteins (Fig. 2b). The interaction between 3xFLAG MCPIP3, KRT14 and 14-3-3σ was confirmed by western blot analysis of 3xFLAG-MCPIP3 immunoprecipitates (Fig. 2c).

### Silencing of MCPIP3 and KRT14 advances the cell cycle from S to M phase

**Fig. 3:**
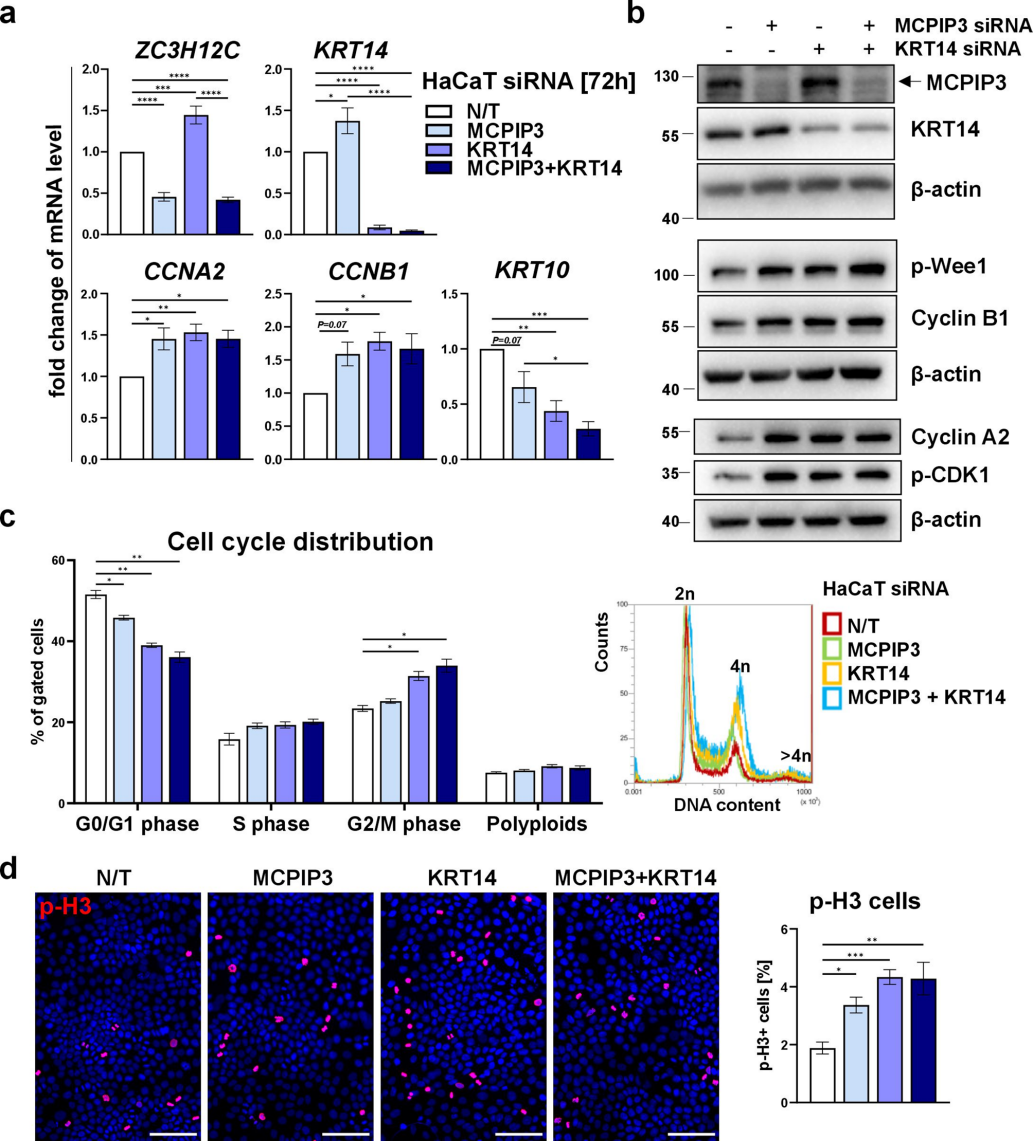
Silencing of MCPIP3 and KRT14 modulates the cell cycle distribution of HaCaT cells.** a** RT-qPCR analysis of *ZC3H12C*, *KRT14*, *CCNA2*, *CCNB1* and *KRT10* levels in HaCaT cells transfected with indicated siRNA for 72 h. *EF2* was used as a reference gene. **b** Representative western blot analysis of MCPIP3, KRT14, p-WEE1, Cyclin B1, Cyclin A2, p-CDK1 and β-actin. **c** The cells were stained with PI and analysed by flow cytometry. Representative flow cytometry plot is shown. Graph represents quantification of the percentage of cells within each stage of the cell cycle. **d** Representative image of p-H3.3 staining and quantification of the percentage of p-H3—positive cells. Data are shown as means and SEM from at least 3 independent experiments. Statistical significance was calculated by one-way (**a**, **d**) or two-way ANOVA (**c**). *P < 0.05, **P < 0.01, ***P < 0.001, ****P < 0.0001.

We next investigated the effects of transient expression of siRNA specific to MCPIP3, KRT14 or both, on HaCaT cells proliferation and progression through the cell cycle. Efficient reduction of MCPIP3 and KRT14 in siRNA treated cells was confirmed by RT-qPCR and western blot analyses (Fig. 3a, b).

The effect of gene knockdown on the expression of key modulators of the cell cycle was next investigated. The mRNA and protein expression of S/G2 and G2/M phase specific cyclins, cyclin A2 and cyclin B1 respectively, was increased in both MCPIP3- and KRT14- and double MCPIP3-/KRT14- silenced cells (Fig. 3a, b). High abundance of phosphorylated Wee1 (p-Ser642) indicative of activity and the level of inhibitory phosphorylation on Cdk1 (p-Y15), thus critical regulators of G2/M progression, were also observed in these cells (Fig. 3b).

Propidium iodine staining showed decreased proportion of G0/G1-phase cells, whereas the percentages of S- and G2/M phase populations increased following silencing of MCPIP3 and/or KRT14. The strongest effect was observed in cells silenced for KRT14, which was augmented by additional silencing of MCPIP3 (Fig. 3c).

Finally, immunofluorescence analysis for a mitotic marker p-H3 indicated that there are more mitotically active cells in MCPIP3-, KRT14- and double MCPIP3-/KRT14- silenced cells (Fig. 3d).

### Expression of MCPIP3 is modulated during the cell cycle

**Fig. 4:**
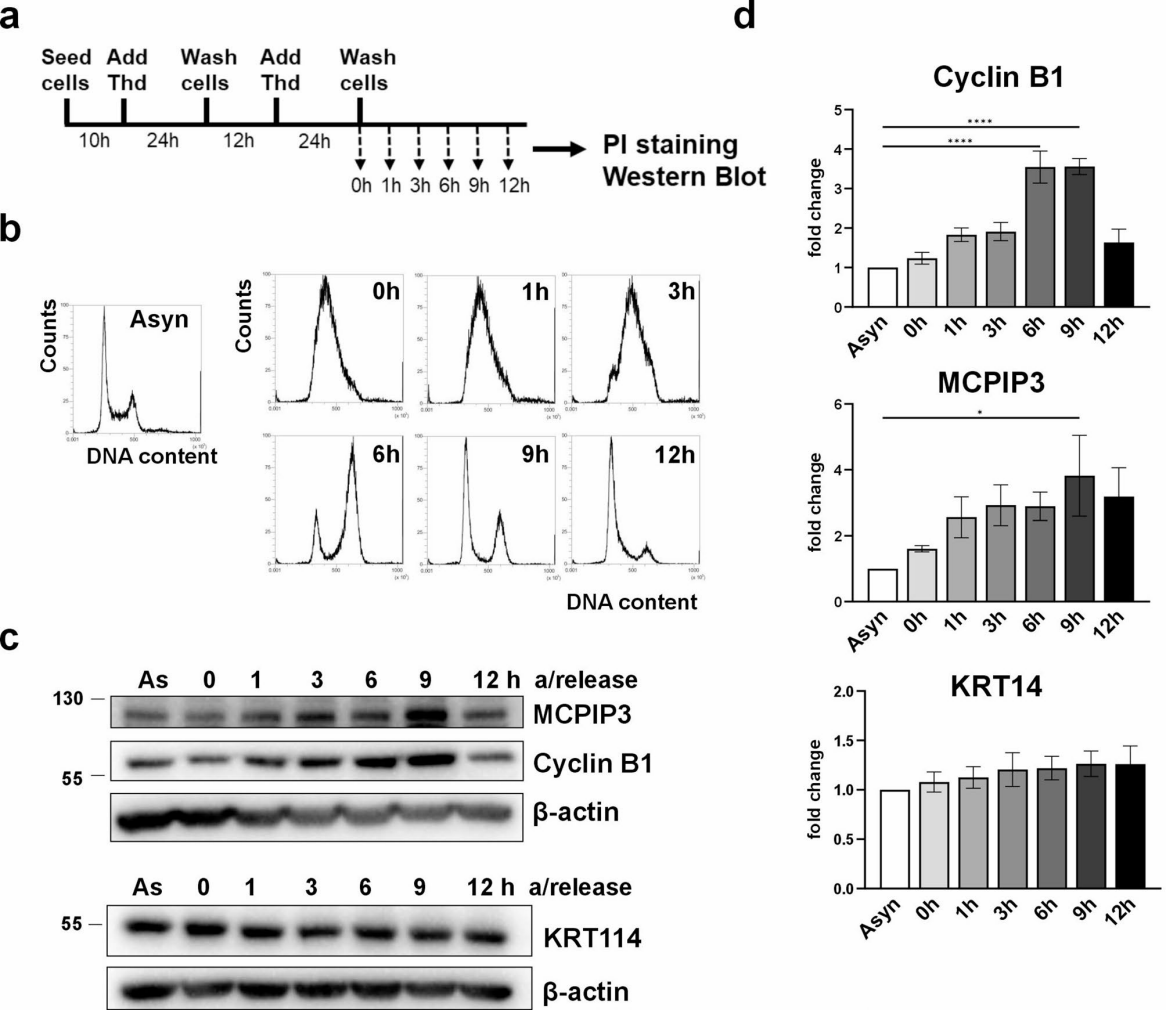
Expression of MCPIP3 fluctuates during the cell cycle of HaCaT cells.** a** A scheme of the experimental pipeline. HaCaT cells were synchronized by a double thymidine block and release protocol, and collected at indicated timepoints for western blot and flow cytometry analyses. **b** The results of flow cytometry analysis of cells stained with PI at different stages of the cell cycle. **c** Representative western blot analysis of MCPIP3, Cyclin B1, KRT14, and β-actin. **d** Densitometric quantification of the western blot data. β-actin was used as a reference protein. Data are shown as means and SEM from 3 independent experiments. Statistical significance was calculated by one-way ANOVA. *P < 0.05, ****P < 0.0001. As/Asyn–asynchronous. Thd–thymidine.

HaCaT cells were synchronized at G1/S boundary by double thymidine block and release protocol. After release, cells at distinct phases of the cell cycle were collected and analysed (Fig. 4a). DNA content in the synchronous cells and in asynchronous controls was analysed by flow cytometry, which indicated efficient synchrony of the cells. After release, the cells progressed through the cell cycle with the following kinetics: 1 h – early S; 3 h – middle S; 6 h – G2; 9 h – G2/M; 12 h – G1 phase (Fig. 4b). Western blot analysis of cyclin B1 confirmed accumulation of this G2/M-phase cyclin at 6 h and 9 h timepoints. The level of MCPIP3 protein also fluctuated during the cell cycle, reaching the highest value at 9 h, corresponding to the highest amount of mitotically active cells. In addition, at this timepoint a small shift on the gel was observed, suggesting MCPIP3 is posttranslationally modified (by phosphorylation or other modification). We also verified that the protein level of KRT14 is not modulated during progression of the cell cycle in HaCaTs (Fig. 4c, d).

## Discussion

MCPIP family proteins are modulators of inflammatory response and also other cellular processes. The role of MCPIP1 in cell differentiation, apoptosis, angiogenesis and carcinogenesis has been documented in many publications^24–28^. In contrast, the function of MCPIP3 in this context has been much less investigated. MCPIP3 was previously described as a potential tumor suppressor gene in colorectal cancer^29^. We speculate that deregulated expression of MCPIP3 in cancerous tissue would promote or enhance abnormal expression of cell-cycle modulators, thus affecting cell proliferative activity. In the skin, our recent study described MCPIP3 as a novel modulator of epidermal proliferation and differentiation equilibrium, which suggested its role in the cell cycle regulation^7^.

In the present study, we discovered that the protein levels of MCPIP3 oscillate during the cell cycle of HaCaT keratinocytes, reaching the highest value in late G2 or mitotic cells, 9 h after release from a double thymidine block. Interestingly, MCPIP3 follows a somewhat similar pattern of expression as cyclin B1, the key modulator of the G2/M transition. In mammalian cells, the level of cyclin B1 gradually increases from S to G2 phases, peaks in late G2 and early M phase, and is rapidly degraded after metaphase^30,31^. High expression level of MCPIP3 in peri-mitotic cells suggests its involvement in the processes regulating cell division.

In agreement with these observations, silencing of MCPIP3 in HaCaTs resulted in an elevated levels of cell cycle activity, manifested by increased levels of S/G2, G2/M and mitotic phase markers, namely Cyclin A2, Cyclin B1, and pSer10-H3. An analysis of DNA content by flow cytometry indicated reduced population of diploid cells (DNA = 2n) in G0/G1 phase, with increased percentages of S/G2/M phase cells (2n < DNA < = 4n), also suggesting higher cell proliferation rate.

We previously demonstrated that MCPIP3 negatively regulates the level of cyclin B1. The mechanism involves a direct nucleolytic cleavage within the 3’UTR sequence of the corresponding *CCNB1* transcript^7^. Our results also indicated that MCPIP3 negatively regulates the mRNA of cyclin A2, as the *CCNA2* transcript level was significantly upregulated in MCPIP3-silenced cells. As a result, silencing of MCPIP3 upregulates the protein levels of cyclin A2 and cyclin B1.

In the cell cycle, expression of cyclin A2 and cyclin B1 is regulated at both transcriptional and translational levels^32^. It was shown that inhibition of transcription in mitotically arrested cells leads to downregulation of cyclin B1 mRNA and protein^33^. We propose that high level of MCPIP3 RNase in peri-mitotic cells is a part of the mechanism that contributes to the regulation of cyclin B1 mRNA. Our results have shed light on the complex role of the post-transcriptional gene expression in the regulation of cell cycle and should be further explored.

One of the limitations of our study is the use of HaCaT cells, spontaneously immortalized human keratinocytes^17^. The HaCaT cell line has preserved high differentiative potential and thus it is a good model of skin keratinocytes^34,35^. Similarly to primary keratinocytes, HaCaT cells display constitutive as well as inducible expression of keratins and other differentiation markers^36,37^. However, HaCaT cells contain gain-of-function mutations in the *TP53* gene^38^, which suggests that data obtained in these cells should be interpreted with caution. Nevertheless, as noncancerous cells, HaCaTs are widely used especially to study processes related to keratinocyte proliferation and differentiation. In contrast to HaCaTs, the NHEK cells would not allow conducting long-term experiments, because their proliferation and differentiation properties change as the passage number increases, and these cells eventually undergo cornification upon *in vitro* differentiation. Moreover, the donor-to-donor variability limits the comparability of experimental results of studies employing NHEKs. Thus, we have used HaCaT cells as a robust alternative to NHEKs, but we emphasize that this is a model and the conclusions of our study should be further strengthened by follow-up investigations of primary keratinocytes.

In the proposed mechanism, MCPIP3 regulates cell cycle related events indirectly, *via* negative regulation of transcripts encoding cell cycle related proteins, and also *via* direct interaction with cell cycle regulatory proteins. The immunoprecipitation-proteomics approach revealed that MCPIP3 forms complexes with KRT5 and KRT14, intermediate filament proteins typically expressed in the basal keratinocytes characterized by a high proliferative activity^39^, with desmoplakin, a component of desmosome involved in anchoring the filaments^40^, and with “stress-related keratins” namely KRT17 and KRT6A (Fig. 2a)^41^. Furthermore, we observed that MCPIP3 interacts with regulators of cell polarity, such as microtubule affinity regulated kinases MARK1 and MARK3 (**Supplemental Table S3**)^42^.

Our analysis revealed that highly abundant protein family present in MCPIP3 immunoprecipitates included almost all known isoforms of 14-3-3 members: 14-3-3γ, 14-3-3ε, 14-3-3ζ, 14-3-3η, 14-3-3θ and 14-3-3σ^43,44^. In a recent study, the 14-3-3 proteins were also identified in MCPIP3 immunoprecipitates in mast cells^45^. Also, the 14-3-3 isoforms (except 14-3-3σ) were described as interactors of MCPIP1 in HeLa cells, affecting protein stability^46^. It was demonstrated that MCPIP1 interacts with 14-3-3 proteins upon stimulation with IL-1β or TLR ligands, and this interaction is mediated by phosphorylation of MCPIP1 by IRAK1.

We speculate that in our model the mechanism of complex formation is most likely based on a direct interaction between MCPIP3 and 14-3-3 members. According to bioinformatic predictions, MCPIP3 protein contains high-scoring potential 14-3-3 binding sites^47^, with RXpSXP and RXXXpSXP typical consensus sequences^47,48^. We validated the interaction between 3xFLAG-MCPIP3, KRT14 and 14-3-3σ by western blot analysis of 3xFLAG-MCPIP3 immunoprecipitates.

The 14-3-3 proteins are a family of phospho-binding proteins that play particularly important function in the cell cycle control^49^. Notably, 14-3-3 proteins bind several keratins during progression through the cell cycle also and regulate intermediate filament network reorganization in response to mechanical forces during embryogenesis^50–52^. Of them, the 14-3-3σ isoform is particularly important for the maintenance of the G2/M checkpoint. Its expression is rapidly upregulated by p53 and its high expression level ensures that DNA damaged cells maintain in a G2/M arrested state^53^. The interaction between MCPIP3 and 14-3-3σ isoform may be a mechanism contributing to the stabilization of MCPIP3 protein in late G2/M phase cells. Our data indicate that the level of MCPIP3 protein reaches its highest level in peri-mitotic cells. Further studies are however needed to fully differentiate whether MCPIP3 activity is required for late G2 events, mitosis or for the G2/M checkpoint. We speculate that MCPIP3 undergoes specific phosphorylation that promotes 14-3-3σ binding, thus increasing its stability at G2/M phase. Consistent with this hypothesis, a small electrophoretic shift was observed in cell released from a double thymidine block for 9 h, which could be due to phosphorylation. However, an identification of the specific kinase responsible for phosphorylation remains challenging because standard IP-MS-based methods often miss transient kinase-substrate interactions.

We propose that MCPIP3 plays a dual role at G2/M phases of the cell cycle: directly by the control of the mRNA stability, and also indirectly *via* forming immune complexes with some important modulators of cell proliferation and differentiation. Mechanistically, most likely 14-3-3σ (or other 14-3-3 member) functions as a scaffold that associates with MCPIP3 and keratin 17 (and its polymerization partner keratin 6 A^54^, enabling complex formation. Indeed, it has been demonstrated that the interaction between 14-3-3σ stabilizes a complex involving keratin 5 and keratin 17, regulating cell motility and invasion through actin and intermediate filament dynamics^55^. We speculate that the interaction with keratin 6 A and 17 could be important for the regulation of proliferation and growth of keratinocytes, and possibly may have implications in carcinogenesis. It was previously demonstrated that keratin 17 positively regulates cell growth and proliferation. KRT17-deficient keratinocytes are smaller, with reduced activity of the mTOR/AKT signaling pathway essential for cell growth and protein synthesis^41,56^. In addition, genetic ablation of KRT17 delays tumor initiation and growth and reduces keratinocyte proliferation in basaloid skin tumors^57^. These findings suggest that the role of MCPIP3 in skin carcinogenesis should be further investigated.

Apart from the regulation of the G2/M checkpoint, 14-3-3σ has been implicated in the regulation of epithelial cell polarity^58^, and the interaction between KRT14 and 14-3-3σ was showed to be compulsory for the initiation of the keratinocyte proliferation to differentiation switch^5,59^. A direct effect of KRT14 on epithelial cell proliferation has been demonstrated in recent studies. Specifically, mice with knock-in mutation that promoted disruption of KRT14 dependent disulfide bonding exhibited enhanced proliferation and altered differentiation pattern of epidermis^59^. In addition, KRT14 knockout in murine small airway epithelial cells resulted in an increased clonogenicity^60^. In agreement with these observations, we found that silencing of KRT14 accelerates the cell cycle activity of HaCaT cells. We further discovered that silencing of KRT14 on top of MCPIP3 leads to even higher rate of cell division and proliferation, as compared to silencing of KRT14 or MCPIP3 alone.

As we previously demonstrated, keratinocyte-specific deletion of MCPIP3 in mouse skin (MCPIP3^EKO^ model) leads to an uncontrolled cell cycle activity, increased epidermal proliferation rate and abnormal differentiation pattern^7^. Presumably, in this model the disruption of MCPIP3-KRT14 protein complexes in the basal epidermal compartment is one of the plausible mechanism contributing to the hyperproliferative phenotype of MCPIP3^EKO^ epidermis.

Altogether, our study implies that MCPIP3 RNase modulates cell cycle related events, particularly those related to the G2/M phase transition. It also emphasizes the importance of studying the role of the post-transcriptional control of gene expression in cell cycle regulation.

## Supplementary Information

Below is the link to the electronic supplementary material.

Supplementary Material 1

Supplementary Material 2

## Data Availability

The data underlying this article is provided within the manuscript and supplementary information. Proteomics data are available via ProteomeXchange with identifier PXD067727.
