# Supplementary material for: MCPIP3/Regnase-3 binds 14-3-3 proteins and contributes to the regulation of the cell cycle in human immortalized keratinocytes
Source: Sci Rep. 2025 Sep 26;15:33222. doi: 10.1038/s41598-025-18468-y (PMC12475053; doi:10.1038/s41598-025-18468-y)
Supplement: Supplementary file 2 — Supplementary Material 2 [file 41598_2025_18468_MOESM2_ESM.docx]

| **Gene** | **Starter For (5’->3’)** | **Starter Rev (5’->3’)** |
| --- | --- | --- |
| ***EF2*** | GACATCACCAAGGGTGTGCAG | TCAGCACACTGGCATAGAGGC |
| ***CCNA2*** | GGTACTGAAGTCCGGGAACC | CAGGGCATCTTCACGCTCTA |
| ***CCNB1*** | CCTCTCCAAGCCCAATGGAA | ACTTCCCGACCCAGTAGGTA |
| ***KRT10*** | AGTCCCAACTGGCCTTGAAAC | TGCACACAGTAGCGACCTTC |
| ***KRT14*** | CCAGCTCAGCATGAAAGCATC | TGAGATCCAGAGGAGAACTG |
| ***ZC3H12C*** | AGAGGTCTGAATCTCCAATGCAA | CGGGATTGCTCTTTCCTCCAA |

**Supplementary Table S1. Sequences of primers used in the study.**

**Supplementary Table S2. List of antibodies used for western blot (WB) or immunofluorescence (IF).**

| **Antigen** | **Host species** | **Dilution/application** | **Catalogue number** | **Company** |
| --- | --- | --- | --- | --- |
| **KRT14** | Rabbit | 1:1000 (WB) | 905304 | Biolegend, San Diego, CA, USA |
| **MCPIP3** | Rabbit | 1:1000 (WB) | GTX85195 | GeneTex Irvine, CA, USA |
| **Cyclin A2** | Mouse | 1:2000 (WB) | 4656 | Cell Signaling Technology |
| **Cyclin B1** | Rabbit | 1:1000 (WB) | 12231 |  |
| **p-CDK1 (Tyr15)** | Rabbit | 1:1000 (WB) | 4539 |  |
| **p-Histone H3 (Ser10)** | Rabbit | 1: (IF) | 3377 |  |
| **p-Wee1 (Ser642)** | Rabbit | 1:1000 (WB) | 4910 |  |
| **β-actin** | Mouse | 1:2000 (WB) | A1978 | Sigma Aldrich |
| **rabbit IgG, HRP-linked** | Goat | 1:20000 | A0545 |  |
| **mouse IgG, HRP-linked** | Goat | 1:20000 | 554002 | Pharmingen, San Diego, CA, USA |
| **Alexa Fluor 546 anti-rabbit** | Goat | 1:500 (IF) | A11035 | Invitrogen, Darmstadt, Germany |
